# Clinical significance and molecular mechanism of angiotensin-converting enzyme 2 in hepatocellular carcinoma tissues

**DOI:** 10.1080/21655979.2021.1952791

**Published:** 2021-08-08

**Authors:** Wei-Jian Huang, Wei-Ying He, Jian-Di Li, Rong-Quan He, Zhi-Guang Huang, Xian-Guo Zhou, Jian-Jun Li, Da-Tong Zeng, Ji-Tian Chen, Wei-Zi Wu, Yi-Wu Dang, Gang Chen

**Affiliations:** a Department of Pathology, Redcross Hospital of Yulin, Yulin, P.R. China; b Department of Pathology, First Affiliated Hospital of Guangxi Medical University, Nanning, P.R. China; c Department of Medical Oncology, First Affiliated Hospital of Guangxi Medical University, Nanning, P.R. China; d Research Department, Guangxi Medical University Cancer Hospital, Nanning, P.R. China; e Department of General Surgery, Second Affiliated Hospital of Guangxi Medical University, Nanning, P. R. China; f Department of Pathology, Lingshan People’s Hospital, Qinzhou, P.R. China

**Keywords:** *ACE2*, hepatocellular carcinoma, COVID-19

## Abstract

During the pandemic of the coronavirus disease 2019, there exist quite a few studies on angiotensin-converting enzyme 2 (*ACE2*) and SARS-CoV-2 infection, while little is known about *ACE2* in hepatocellular carcinoma (HCC). The detailed mechanism among *ACE2* and HCC still remains unclear, which needs to be further investigated. In the current study with a total of 6,926 samples, *ACE2* expression was downregulated in HCC compared with non-HCC samples (standardized mean difference = −0.41). With the area under the curve of summary receiver operating characteristic = 0.82, *ACE2* expression showed a better ability to differentiate HCC from non-HCC. The mRNA expression of *ACE2* was related to the age, alpha-fetoprotein levels and cirrhosis of HCC patients, and it was identified as a protected factor for HCC patients via Kaplan–Meier survival, Cox regression analyses. The potential molecular mechanism of *ACE2* may be relevant to catabolic and cell division. In all, decreasing *ACE2* expression can be seen in HCC, and its protective role for HCC patients and underlying mechanisms were explored in the study.

## Graphical Abtstract

**Figure:**
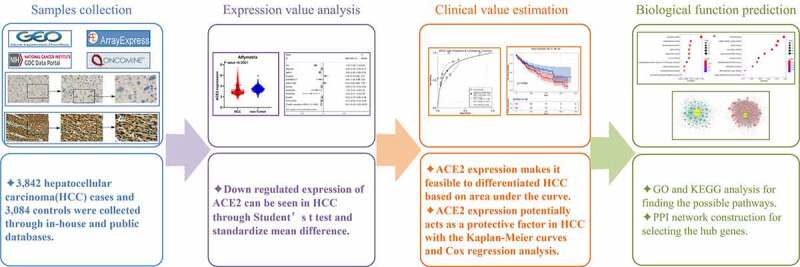

## Introduction

Cancer is a serious worldwide public health threat. During the pandemic of the coronavirus disease 2019 (COVID-19), cancer patients are reported to be easier to infect SARS-CoV-2 and have a higher risk of severe events[1]. Taking the United States as an example, liver cancer mortality ranks fourth among males and seventh among females, with a five-year relative survival rates of only 20%[2]. Recent reports have demonstrated that especially in females, the incidence is still increasing by 2% per year while it has been temporarily steady in males[2]. Hepatocellular carcinoma (HCC) is the major subtype of liver cancer [3,4] with complex pathogenesis. In the United States nationally, the recent and ongoing hepatitis C virus (HCV) epidemic poses a threat to the prevention of liver cancer [2], while hepatitis B virus (HBV) infection [4], obesity, excessive alcohol consumption [2], and intake of aflatoxin [5] are also recognized as risk factors for HCC. As for molecular events, multiple studies have confirmed that the abnormal expression of oncogenes plays an important part in the progression of HCC [6–8]. Thus, the mechanism of the occurrence and development of HCC are intricate and in urgent need of clarification.

With the SARS-CoV-2 (COVID-19) pandemic, the COVID-19-related angiotensin-converting enzyme 2 (*ACE2*) has attracted worldwide attention because it is a major receptor that allows the virus to enter human cells[9], while few reports about *ACE2* expression and its value in HCC can be seen. *ACE2*, which belongs to the renin-angiotensin-aldosterone system (RAAS), is the homolog of angiotensin-converting enzyme (ACE) [10,11]. As the antagonist of Angiotensin II (Ang II), *ACE2* converts Ang II into Ang (1–7) and meditates vasodilator, anti-inflammatory, anti-fibrosis, anti-apoptosis, and anti-proliferation processes [12–14]. *ACE2* exists in vascular endothelial cells and smooth muscle cells of all organs [15], as well as in tissues rich in glucose, such as liver and adipose tissue[13]. Interestingly, in the digestive and respiratory tracts, *ACE2* expression was found to be almost restrained in epithelial cells, whether in normal or malignant tissues[16]. In tumors, the *ACE2* is considered to be a protective factor against tumorigenesis; the up-regulation of *ACE2* indicates a good prognosis in multiple cancers and is inversely associated with some crucial pathways of tumor progress, such as proliferation and mismatch repair[17]. Studies have shown that in HCC, *ACE2* could serve as a protective factor and its expression was reduced in HCC patients [18–20], and also might improve the survival of patients without vascular invasion[18]; however, some limitations could be seen in these reports, such as small sample size and a lack of validation with in-house clinical samples. Furthermore, little integrated computational analysis based on samples from diverse sources can be seen, where the potential clinical value and molecular mechanism of *ACE2* in HCC were explored.

From the preparatory work, we reasonably assumed that low *ACE2* expression has a closely relation with HCC development. Based on in-house immunohistochemistry (IHC), tissue microarrays and high-throughput datasets from multiple public databases, the current study aims to investigate expression level, clinical values and potential mechanisms of *ACE2* in HCC, which may contribute to clinical management of HCC and understanding of the pathogenesis of this disease.

## Materials and methods

### *The clinical value of* ACE2 *expression in HCC tissues*

#### *Protein and mRNA expression of* ACE2 *in HCC*

##### IHC with in-house samples

The current work was approved by the institutional review board of the First Affiliated Hospital of Guangxi Medical University (No. 2019-KY-Guoji-113). All experiments were conducted according to applicable rules, and informed consent was obtained from all patients. IHC was applied to analyze the protein expression of ACE2. A total of 55 HCC and 71 non-tumor liver samples was included, while 47 HCC and 63 non-tumor liver cases were obtained from Pantomics Company’s tissue array (TMA No. LVC1601, Guilin, P.R. China) and eight pairs of HCC and corresponding nonmalignant counterparts were collected from the First Affiliated Hospital of Guangxi Medical University.

The formalin-immobilized and embedded samples went through a dehydration process, and then endogenous peroxidases were inhibited and the antigen was exposed. A two-step IHC technique was applied. Anti-ACE2 antibody (ab, rabbit polyclonal to ACE2, Abcam) was used as the first antibody in the experimental group, and phosphate-buffered saline (PBS) was used as a comparison taking the place of the first antibody. Both were incubated overnight at 37°C. Then, the second antibody was added to the samples at 25°C, which was incubated for half an hour. After hematoxylin staining, two pathologists, respectively, evaluated the staining intensity and the percentage of stained positive cells. The score of ACE2 protein expression was the product of staining strength score and score of percentage of positive stained cells, as we previously reported [21].

##### Collection of the public datasets

Using the search terms ‘hepatocellular carcinoma’ and ‘HCC’, the Gene Expression Omnibus, ArrayExpress [22], Sequence Read Archive, Oncomine, The Cancer Genome Atlas (TCGA), and PubMed databases were searched for high-throughput datasets. In this study, each dataset was considered for further analyzed only when it met the following inclusion criteria: 1) *Homo sapiens*-related studies; 2) containing both HCC and non-HCC samples; 3) including *ACE2* mRNA expression data. Exclusion criteria were as follows: 1) the sample number of HCC or non-HCC <3; 2) containing duplicate or unavailable data. The Genotype-Tissue Expression (GTEx) project was also used to expand normal samples and was combined with TCGA.

Log_2_(X+1) transformation (X means the raw data of gene expression) was undertaken for gene levels of the public datasets. Microarrays from Gene Expression Omnibus with the same platform, such as ‘Affymetrix’, were combined for further analysis. For combined microarrays, the batch effect was eliminated through surrogate variable analysis [23] package in R (v 4.02), and *ACE2* expression was finally measured on the basis of the above datasets.

##### ACE2 *expression in HCC and non-HCC samples*

To obtain a stronger result, in-house IHC and high-throughput datasets were combined in order to increase the number of cases. Computed with Stata 15.0 (StataCorp LLC, College Station, TX, USA), the standardized mean difference (SMD) and summary receiver operating characteristic (SROC) were the two major indices used to measure the expression and discriminating ability of *ACE2* in HCC. The model selected was dependent on the *I^2^* value of *I^2^* test, and random effect model was chosen when *I^2^* > 50%, as it indicates significant heterogeneity. Moreover, jitter plots and receiver operating characteristic (ROC) curves were drawn with GraphPad Prism 8. Finally, the *Egger’s* diagram and *Deek’s* funnel plot were applied to measure publication bias.

#### *Clinical value and prognosis prediction ability of* ACE2 *in HCC*

Clinical parameters, containing gender, age, vital status, Tumor Node Metastasis (TNM) stage, pathological stage, cirrhosis, alpha-fetoprotein (AFP) level, HBV infection, HCV infection, alcohol consumption, and vascular invasion, were collected from the IHC samples and TCGA database. For assessing the correlation between *ACE2* and clinical features of HCC patients, *student’s t* test and analysis of variance was chosen to compare *ACE2* expression between the subgroups of clinical features. *p* < 0.05 represented statistical significance.

In order to evaluate the prognostic capacity of *ACE2*, survival status, overall survival (OS), and recurrence-free survival (RFS) time were all collected from the above datasets. In a dataset, HCC patients were divided into two groups, those with less than median *ACE2* expression level were classified to low-*ACE2* expression group, while the remaining HCC patients were classified into the high-*ACE2* expression group. Kaplan–Meier survival and univariate Cox regression analyses were then performed, while multivariate Cox regression analysis was based on clinical characteristic with a *p* < 0.05 in univariate Cox regression. Moreover, in the computation of the pooled hazard ratio (HR), which is essential for measuring the prognosis prediction capabilities of *ACE2* expression, studies that met the above requirements with prognostic information were searched for and included.

### *Selection of* ACE2*-related upregulated expression genes (UEGs) and downregulated expression genes (DEGs)*

To screen the genes that were closely associated with the mechanism of *ACE2* expression in HCC, *Pearson’s* correlation analysis was performed based on the datasets above. Genes with both absolute value of *Pearson’s* correlation coefficient ≥ 0.3 and *p* < 0.05 were identified as *ACE2*-related genes. UEGs were selected through the threshold Log_2_ fold change >1 and p<0.05 (based on the limma package [24] in R) and the 95% confidence interval of SMD without zero; while DEGs were those with the threshold Log_2_ fold change <1 and p<0.05 and the 95% confidence interval of SMD without zero. *ACE2*-related UEGs were the intersection of *ACE2*-related genes (those with *Pearson’s* correlation coefficient ≥0.3) and UEGs, while *ACE2*-related DEGs were the intersection of *ACE2*-related genes (those with *Pearson’s* correlation coefficient <−0.3) and DEGs.

### *Potential mechanism analysis of* ACE2 *in HCC*

Based on *ACE2*-related UEGs and DEGs, Gene Ontology (GO) annotation and the Kyoto Encyclopedia of Genes and Genomes (KEGG) were applied to assess the possible items and pathways in which *ACE2* might involve. Protein–protein interaction (PPI) networks were exploited through The Search Tool for the Retrieval of Interacting Genes database, and then the hub genes of *ACE2*-related UEGs and DEGs were screened in the cytoHubba plugin in Cytoscape (v3.5.0) with the highest degree centrality. The subnetwork of PPI consisted of some *ACE2*-related DEGs, where *ACE2* involved, was selected for further exploring the molecular functions (in GO analysis) of *ACE2* in HCC.

## Results

### The short summary of the results

Little was known about the important role of *ACE2* in HCC, which was exploited in the study. Both mRNA and protein levels of *ACE2* were significantly lower in HCC based on 6,926 samples. *ACE2* expression made it feasible to differentiate HCC via AUC. The mRNA expression of *ACE2* was related to some clinical parameters of the HCC patients, containing age, AFP level and cirrhosis. High-*ACE2* expression is found to be associated with the considerable prognoses of HCC patients. Chemical carcinogenesis and the cell cycle were potential pathways that ACE2 involved in HCC.

### *Expression and clinical value of* ACE2 *in HCC*

#### ACE2 *protein expression with in-house samples*

**Figure 1.**
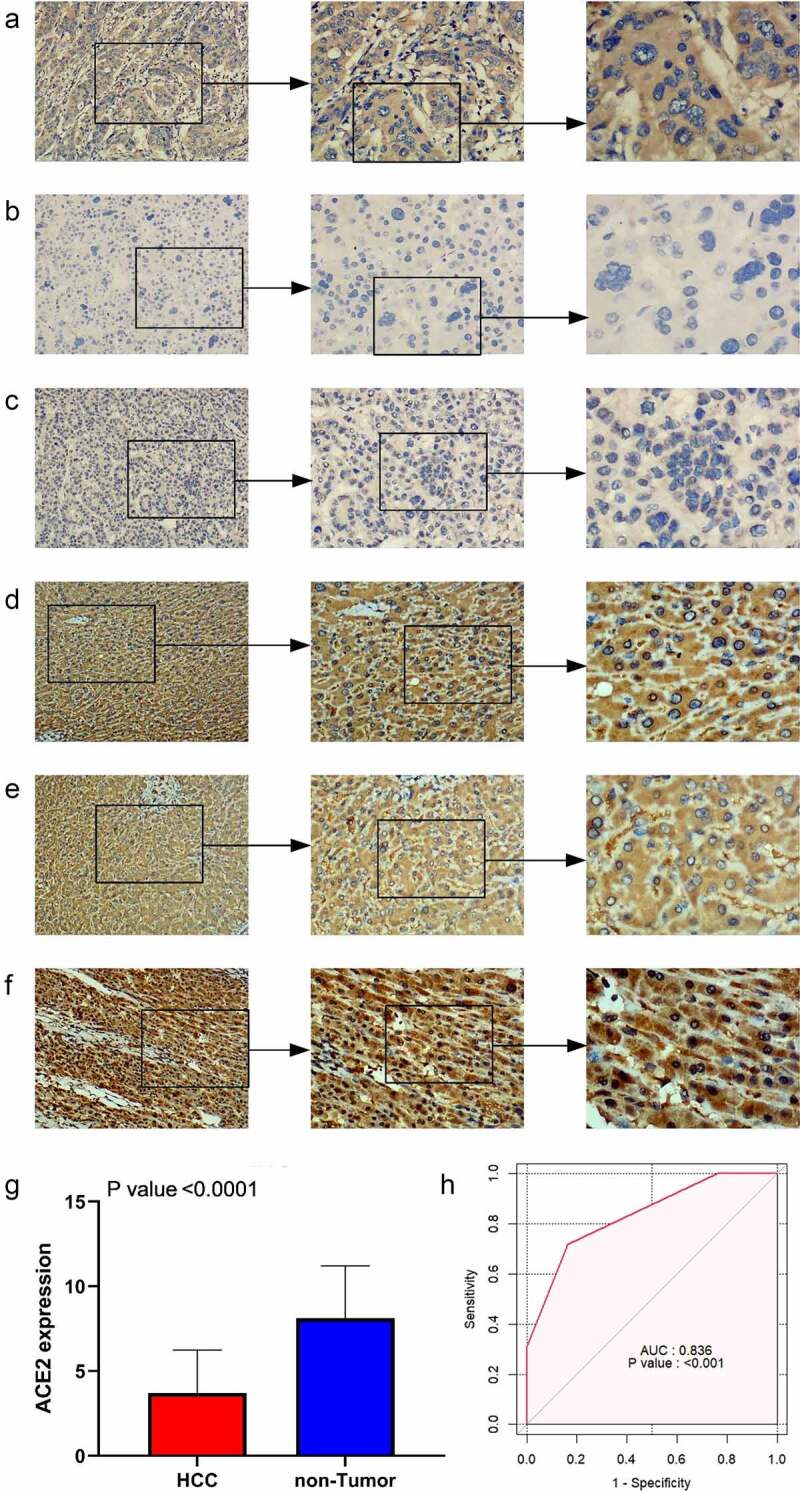
The expression level of *ACE2* protein in hepatocellular carcinoma (HCC) and non-tumor liver tissues based on immunohistochemistry. Panels a-c: representative images of *ACE2* protein expression in HCC tissues. Panels d-f: representative images of *ACE2* protein expression in non-tumor normal liver tissues. In panels a-f, the magnifications of the three images of each panel are 100, 200, and 400 respectively. Panel g: *ACE2* protein expression. Panel h: receiver operating characteristic (ROC) curve with area under the curve (AUC) of *ACE2* protein expression in HCC tissues

ACE2 expression in HCC samples was manifestly downregulated compared with non-tumor cases (Figure 1), and the positive of stained signaling of ACE2 can mainly be seen in the cytoplasm of HCC cells rather than non-HCC hepatocytes. The area under the curve (AUC) of downregulated ACE2 expression to identify HCC was 0.836, indicating the considerable ability of ACE2 expression to distinguish HCC from non-HCC tissues.

#### *Integrative analysis of* ACE2 *mRNA expression in HCC and non-HCC samples*

**Figure 2.**
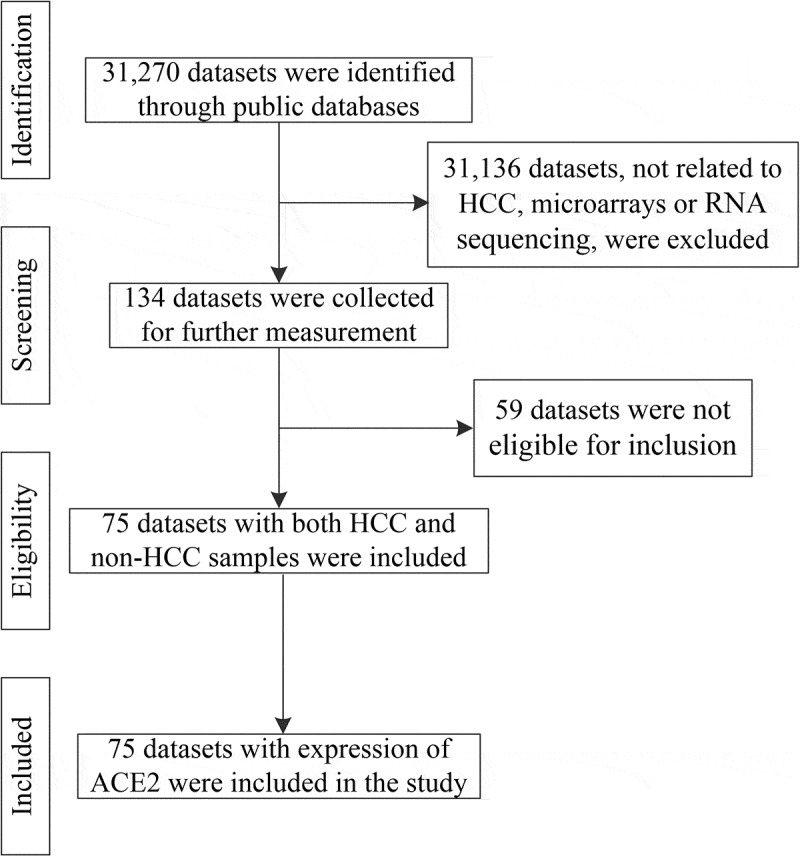
The flow chart of the recruitment procedure for the high-throughput datasets

**Figure 3.**
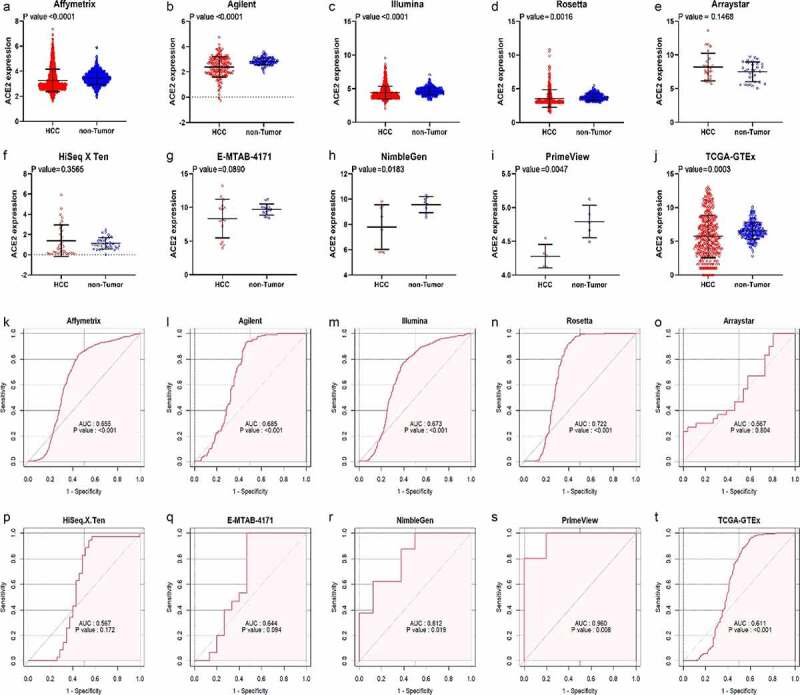
Jitter plots and receiver operating characteristic (ROC) curves of all high-throughput datasets from public databases. Panels a–j the jitter plots of the 10 datasets. Panels k–t: receiver operating characteristic (ROC) curves of the 10 mRNA datasets

After in-house IHC and high-throughput datasets were combined, a total of 3,842 HCC cases and 3,084 controls were involved in the comprehensive analysis of *ACE2* expression in HCC. Figure 2 shows the recruitment procedure for the high-throughput datasets; details about the 10 high-throughput datasets and results from IHC is shown in Table 1. From the jitter plots (Figure 3(a–j)) and Table 1, except for Arraystar, HiSeq X Ten, and E-MTAB-4171, the majority of the studies included showed downregulated expression of *ACE2* in HCC with *p* < 0.05. As for ROC curves (Figure 3(k–t)), seven of ten mRNA datasets all showed AUC > 0.6 (*p* < 0.05).

**Table 1.** Details of IHC result and the 10 high-throughput datasets from public databases with corresponding *ACE2* expression level, AUC of the ROC curve, sensitivity and specificity of *ACE2* in HCC

Datasets	HCC			Non-HCC			ROC	
	N	Mean	SD	N	Mean	SD	Sensitivity	Specificity
Affymetrix	1656	3.261	0.894	1356	3.451	0.501	0.546	0.836
Agilent	186	2.387	0.807	131	2.830	0.278	0.543	0.931
Arraystar	26	8.190	2.051	30	7.496	1.462	1.000	0.233
E-MTAB-4171	15	8.346	2.870	15	9.705	0.829	0.533	1.000
HiSeq X Ten	35	1.386	1.560	35	1.126	0.560	0.457	0.943
Illumina	1117	4.406	0.951	822	4.629	0.516	0.603	0.774
NimbleGen	8	7.785	1.770	8	9.562	0.642	0.875	0.625
PrimeView	5	4.280	0.174	5	4.794	0.240	1.000	0.800
Rosetta	368	3.558	1.308	386	3.784	0.475	0.609	0.917
TCGA-GTEx	371	5.740	3.131	225	6.545	1.238	0.412	0.947
Tissue microarrays	55	3.709	2.536	71	8.113	3.096	0.836	0.718

Notes: HCC, hepatocellular carcinoma; ROC, receiver operating characteristic; N, number; SD, standard deviation.

**Figure 4.**
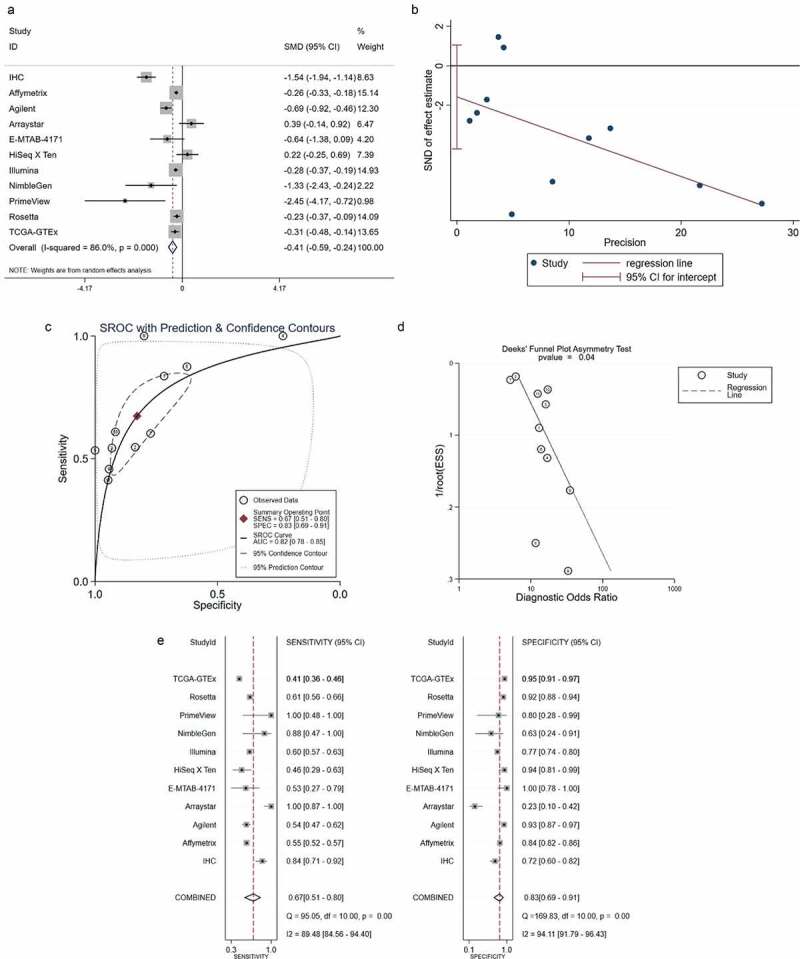
Integrated analysis of the 10 high-throughput datasets and in-house immunohistochemistry (IHC). Panel a: forest plot. Panel b: Egger diagram. Panel c: summary receiver operating characteristic (SROC) curve. Panel d: *Deek’s* funnel plot, Panel e: chart of the sensitivity and specificity

The total SMD displayed by the forest plot was −0.41 (95% confidence interval: −0.59 to −0.24), which further verified the reduced expression of *ACE2* in HCC (Figure 4(a)). Little publication bias of the SMD was found based on the *Egger’s* analysis (*p* = 0.208, Figure 4(b)).

#### *Clinical values of* ACE2 *in the progression of HCC*

The AUC of the SROC was 0.82 (95% confidence interval: 0.78 to 0.85) and indicated favorable distinguishing ability (Figure 4(c–d)). The combined sensitivity and specificity are shown in Figure 4(e) and were 0.67 and 0.83, respectively.

Although no clinical features appeared statistically significant, Node stage with *p* = 0.058 might be considered a related factor to *ACE2* protein expression with in-house IHC analysis (data not shown), which should be further verified in the future.

Based on TCGA dataset (Table 2), cases with higher age (≥60 years old) and cirrhosis possessed higher *ACE2* mRNA expression, while those with poorer vital status – dead, and higher AFP levels (≥400 μg/L) had lower *ACE2* mRNA expression. Accordingly, positive relationships appeared between *ACE2* expression level and age (r [*Pearson’s* correlation coefficient] = 0.148, *p* = 0.004) and cirrhosis (r = 0.186, *p* = 0.006), while negative relationships can be seen between *ACE2* expression value and vital status (r = −0.110, *p* = 0.035) and AFP content (r = −0.177, *p* = 0.03).

**Table 2.** Clinical value of *ACE2* mRNA expression in HCC based on RNA-sequencing data

Clinicopathological features		ACE2 mRNA expression				
		n	mean	SD	P(t)	t/F
Tissue	non-cancer	225	6.545	1.238	<0.001	−4.413
	cancer	371	5.740	3.131		
Gender	male	250	5.901	3.234	0.156	1.423
	female	121	5.409	2.892		
Age	< 60 years	169	5.240	2.996	0.004	2.867
	≥ 60 years	201	6.168	3.192		
Vital status	Alive	240	5.984	3.162	0.035	−2.114
	Dead	130	5.267	3.031		
Tumor stage	T1-T2	275	5.762	3.132	0.572	−0.565
	T3-T4	93	5.551	3.075		
Node stage	N0	252	5.695	3.101	0.178	1.734
	N1	4	2.976	2.306		
	NX	114	5.894	3.174		
Metastasis stage	M0	266	5.568	3.167	0.236	1.449
	M1	4	6.524	2.614		
	MX	101	6.163	3.033		
Pathological stage	Stage I–II	257	5.752	3.141	0.392	−0.856
	Stage III–IV	90	5.424	3.085		
Cirrhosis	No	142	5.702	3.285	0.006	2.750
	Yes	70	6.992	3.062		
AFP content	< 400 μg/L	213	6.097	3.211	0.001	−3.251
	≥ 400 μg/L	65	4.785	2.729		
HBV Infection	No	248	5.921	3.14	0.349	−0.939
	Yes	104	5.576	3.171		
HCV Infection	No	296	5.781	3.140	0.600	0.524
	Yes	56	6.022	3.215		
Alcohol Consumption	No	235	5.894	3.048	0.531	−0.628
	Yes	117	5.670	3.350		
Vascular invasion	No	206	6.029	3.054	0.176	−1.355
	Yes	109	5.531	3.188		

Notes: AFP, alpha-fetoprotein; HBV, hepatitis B virus; HCV, hepatitis C virus.

#### *Prognosis prediction ability of* ACE2 *in HCC*

**Figure 5.**
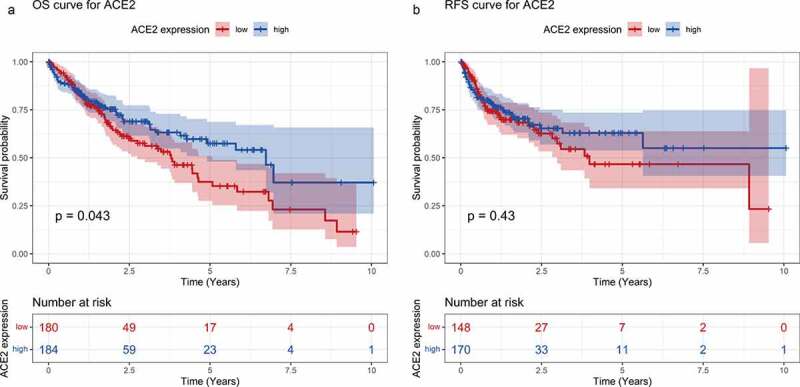
Survival curves for high- and low-*ACE2* expression based of the dataset from The Cancer Genome Atlas. Panel a: overall survival (OS) curve of hepatocellular carcinoma patients. Panel b: recurrence-free survival (RFS) curve of hepatocellular carcinoma patients

As shown in Figure 5(a,b), higher *ACE2* expression predicted better prognosis on overall survival OS time with *p* = 0.043, while recurrence-free survival (RFS) time appeared to have no statistical significance. The total HRs of OS and RFS were 0.80 and 0.84, respectively. Although no significant difference of HR has been found (95% confidence level includes one), a trend can be seen that *ACE2* expression could potentially act as a protective indicator for the survival status of HCC (data not shown). As a result, the prognostic role of *ACE2* needs to be verified with larger numbers of cases and cohorts.

According to the result of the Cox regression analysis shown in Table 3, *ACE2* mRNA expression and HBV infection were the independent influential factors of prognosis for HCC patients based on TCGA RNA-sequencing data.

**Table 3.** Univariate Cox regression and multivariate Cox regression analyses based on the RNA- sequencing data

Characteristics	Univariate Cox regression			Multivariate Cox regression		
	HR	95% CI	*p* value	HR	95% CI	*p* value
ACE2 mRNA expression	0.94	0.88–0.99	0.026	0.92	0.87–0.99	0.017
Age	1.01	1–1.03	0.079	-	-	-
Alcohol consumption	1.02	0.7–1.49	0.898	-	-	-
AFP	1.05	0.64–1.72	0.835	-	-	-
Cirrhosis	0.84	0.48–1.46	0.535	-	-	-
Gender	0.82	0.57–1.16	0.263	-	-	-
HBV Infection	0.36	0.22–0.58	<0.001	0.48	0.28–0.82	0.007
HCV Infection	1.09	0.67–1.78	0.732	-	-	-
T stage	1.63	1.37–1.93	<0.001	1.33	0.6–2.94	0.478
M stage	1.27	1.06–1.53	0.010	1.26	0.97–1.63	0.079
N stage	1.22	1.02–1.47	0.032	0.93	0.71–1.21	0.579
Pathologic stage	1.66	1.35–2.04	<0.001	1.15	0.5–2.61	0.744
Vascular invasion	1.35	0.89–2.05	0.157			

Note: HR, hazard ratio; CI, confidence interval.

### *Screening of the ACE-2 related DEGs and UEGs and their biological functional analyses of* ACE2 *in HCC*

**Figure 6.**
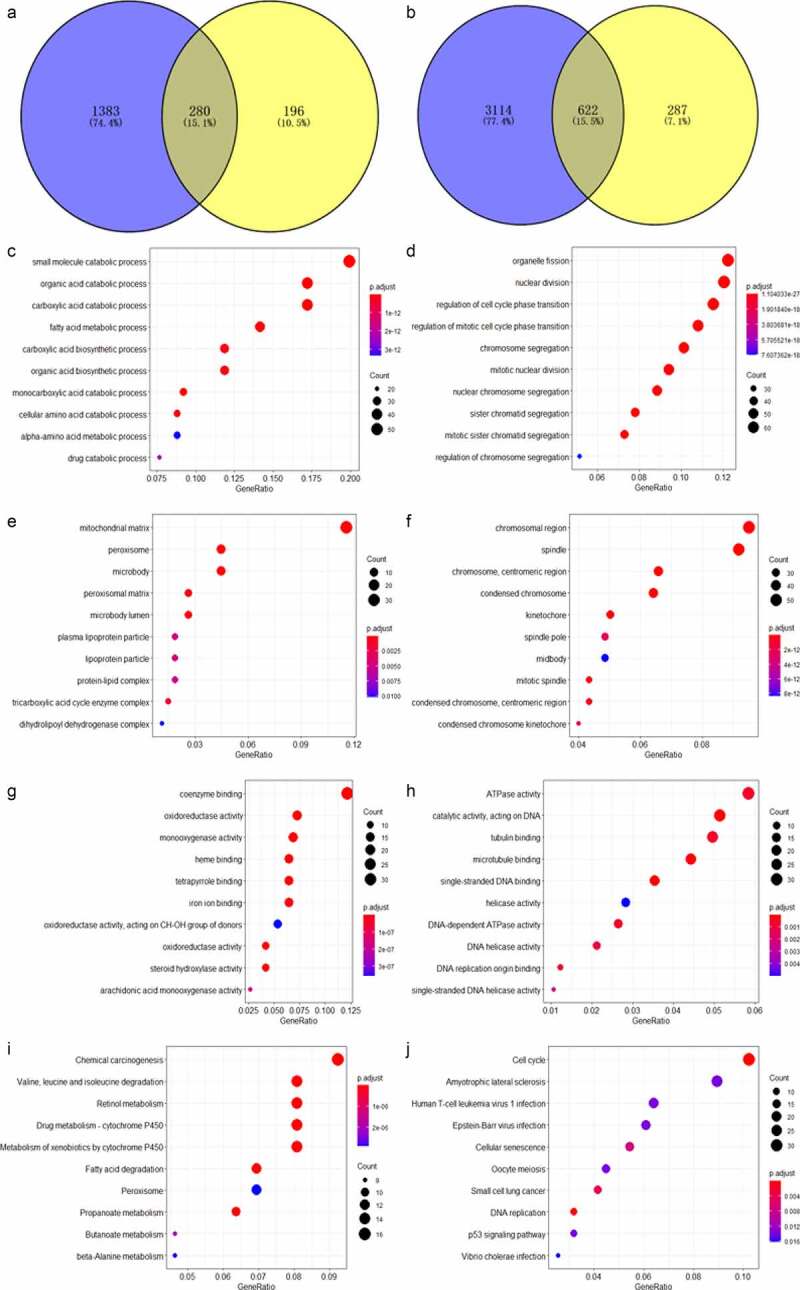
Venn plot showing the result of the two intersections and the result of the Gene Ontology (GO) and Kyoto Encyclopedia of Genes and Genomes (KEGG) analysis visualized by dot plots. The color of the dot represents the *p* value of each enriched item, and the size of the dot reflects the genes enriched to the corresponding items. Panel a: selection of *ACE2*-related downregulated genes. Panel b: selection of *ACE2*-related upregulated genes. Biological processes (panels c–d), cellular compartments (panels e–f) and molecular functions (panel g) of GO analysis based on *ACE2*-related downregulated genes. Panel h: molecular function of GO analysis based on *ACE2*-related upregulated genes. Panel i: KEGG analysis based on *ACE2*-related downregulated genes. Panel j: KEGG analysis based on *ACE2*-related upregulated genes

A total of 280 genes were identified as the *ACE2*-related DEGs (Figure 6(a)), while 622 genes were selected as *ACE2*-related UEGs (Figure 6(b)). The 280 *ACE2*-related DEGs were mostly enriched in small molecule catabolic process, mitochondrial matrix, and coenzyme binding in aspects of biological process, cellular component, and molecular function based on GO enrichment analysis. The 622 *ACE2*-related UEGs were significantly enriched in the gene annotations of organelle fission, chromosomal region, and ATPase activity (Figure 6(c–h)). As for KEGG, chemical carcinogenesis and the cell cycle were the two pathways enriched separately by the *ACE2*-related UEGs and DEGs (Figure 6(i–j)).

**Figure 7.**
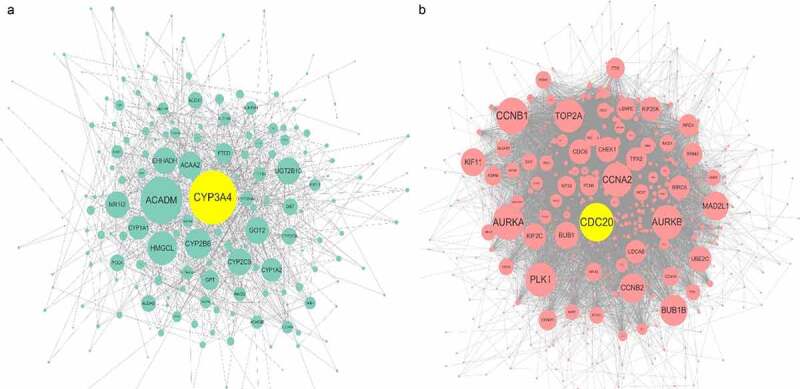
Protein-protein interaction (PPI) network construction of *ACE2*-related upregulated genes and regulated genes. Panel a: PPI of 280 total down-regulated *ACE2*-related genes. Panel b: PPI of 622 total up-regulated *ACE2* related genes. Larger dot size represents greater degree centrality, and the genes in yellow are the hub genes

**Figure 8.**
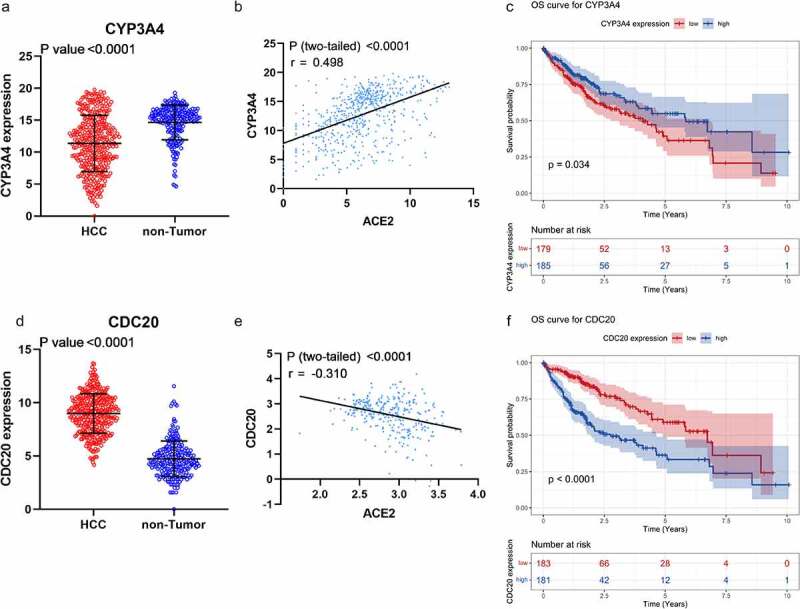
Expression, correlation, and survival analysis of *CYP3A4* and *CDC20* expression. Panel a: the jitter plot of *CYP3A4* expression. Panel b: correlation analysis of *CYP3A4* and *ACE2* expression. Panel c: overall survival curve of *CYP3A4* expression in hepatocellular carcinoma (HCC). Panel d: the jitter plot of *CDC20* expression. Panel e: correlation analysis of *CDC20* and *ACE2* expression. Panel f: overall survival curve of *CDC20* in HCC

**Figure 9.**
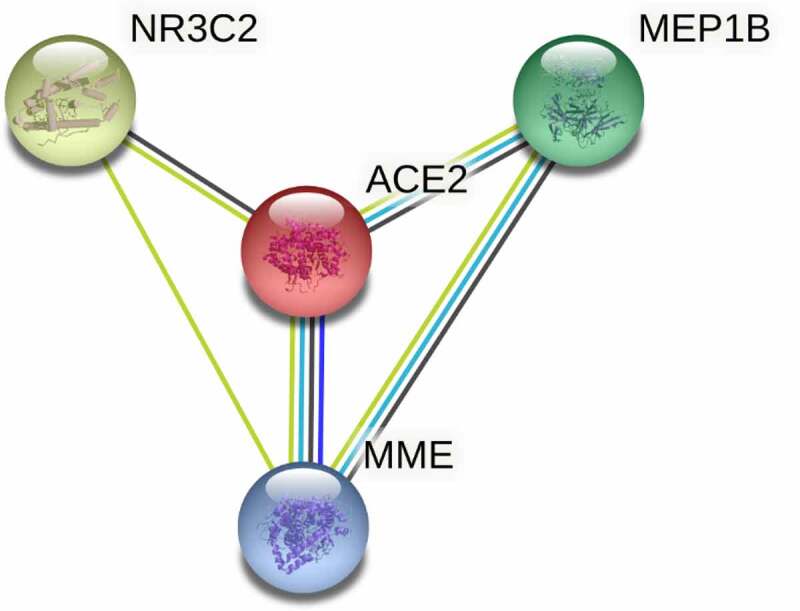
The subnetwork of protein–protein interaction network construction of *ACE2*-related upregulated genes and downregulated genes

In PPI network (Figure 7) analysis, the hub gene *CYP3A4* was selected from *ACE2*-related DEGs due to its highest degree centrality, while the hub gene of *ACE2*-related UEGs was *CDC20*. Low-*CYP3A4* expression and high-*CDC20* expression could be seen in HCC cases (Figure 8(a,d)), and both of the two hub genes were correlated with *ACE2* expression (Figure 8(b,e)) and prognoses of HCC patients (*p* < 0.05, Figure 8(c,f)). According to the GO and KEGG analysis, *CYP3A4* was enriched to the following 13 predicted items: fatty acid metabolic process, carboxylic acid biosynthetic process, organic acid biosynthetic process, drug catabolic process, monooxygenase activity, oxidoreductase activity, steroid hydroxylase activity, heme binding, tetrapyrrole binding, iron ion binding, chemical carcinogenesis, retinol metabolism, drug metabolism – cytochrome P450 and metabolism of xenobiotics by cytochrome P450, while *CDC20* was enriched to the predicted 10 items below: nuclear division, organelle fission, mitotic nuclear division, mitotic sister chromatid segregation, chromosome segregation, regulation of cell cycle phase transition, spindle, cell cycle, Human T-cell leukemia virus 1 infection and oocyte meiosis. Furthermore, the subnetwork (Figure 9) where *ACE2* involved consisted of four *ACE2*-related DEGs – *ACE2, NR3C2, MME*, and *MEP1B*. The GO analysis of the four *ACE2*-related DEGs showed that, *ACE2, MME*, and *MEP1B* clustered in metallopeptidase activity molecular function (data not shown).

## Discussion

As the homolog of angiotensin-converting enzyme, *ACE2* is closely related to COVID-19, but little is known about its important part in HCC. Based on 6,926 samples and various statistical methods, we found that both mRNA and protein levels of *ACE2* were significantly lower in HCC. *ACE2* expression made it feasible to differentiate HCC, which has not been reported in previous studies. The mRNA expression of *ACE2* was related to some clinical parameters – age, AFP level and cirrhosis – of the HCC patients, it was also found to be associated with the prognoses of HCC patients, where the potential clinical value of *ACE2* expression could be seen. Furthermore, we analyzed the potential molecular mechanism of *ACE2* expression in HCC through the *ACE2*-related UEGs and DEGs. In a word, the study reveals the lower expression of *ACE2* and its potential clinical value in HCC, improving the understanding of its prospective molecular mechanism in HCC.

Low-*ACE2* expression in HCC is obvious. Furthermore, a number of studies have reported the down-regulated expression of *ACE2* in various tumors such as breast tumors [25], non-small cell lung cancer [26], pancreatic ductal adenocarcinoma [27], and gallbladder cancer[28]. Reduced expression of *ACE2* was also previously reported in HCC [18–20]; however, there exist some limitations in these reports, such as small sample size (*n* < 500), little data sources and lack of multilevel research. Our study further, based on 6,926 samples from multiple research centers and several statistical methods, reveals significant low-*ACE2* expression in HCC at both the mRNA and protein levels.

Downregulated expression of *ACE2* show quite a few clinical values in HCC. Through *ACE2* expression, HCC patients were likely distinguished from non-HCC patients, indicating its ability to differentiate HCC, which has not been reported before. The mRNA expression of *ACE2* was related to some clinical parameters of the HCC patients, containing age, AFP level and cirrhosis. An impressive finding is that the higher the *ACE2* expression, the lower AFP level, suggesting their negative correlation. Interestingly, AFP is a crucial oncogenic biomarker for HCC, indicating the potential protective role of *ACE2* in HCC. This trend was supported by the OS curve, while *ACE2* expression was further considered a protective and independent prognostic factor for the prognosis of HCC patients via multivariate Cox regression analysis. Thus, the important clinical value of *ACE2* in HCC may include the identification of HCC and its protective effect on HCC patients.

Reduced expression of *ACE2* may take part in tumorigenesis. A report demonstrated that in lung, breast, colon, and pancreatic cancers, *ACE2* was considered an inhibitor of growth, metastasis, and angiogenesis of tumor cells [11]. In breast tumors, the down-regulation of *ACE2* contributes to the invasion and metastasis of breast cancer cells through the store-operated calcium entry (SOCE) and PAK1/NF- κB/Snail1 pathways[14]. In lung cancer, the up-regulation of *ACE2* was considered an inhibitor of cancer progression [26], while its down-regulation was associated with poor prognosis [29]. It can be seen that the down-regulation of *ACE2* plays an important role in tumorigenesis. As a member of the renin-angiotensin-aldosterone system, *ACE2* was reported to be associated with *CD34* expression, VEGF, and the Notch signal pathway, thus participating in angiogenesis in HCC[19]. Based on GO and KEGG analyses, we found that *ACE2*-related UEGs and DEGs were mostly enriched in catabolic and cell division processes, which were both essential processes for carcinogenesis. The possibility of HCC progression due to *ACE2* gene mutation was also considered, but few alterations were found in the mutation spectrum. In conclusion, *ACE2* may be a cancer-related gene involved in the development and progress of HCC.

Through the PPI network, *CYP3A4* and *CDC20* were the two hub genes selected with the highest degree centrality and were associated with the prognosis of HCC patients. *CYP3A4* belongs to cytochrome P450 and participates in the metabolic activation of procarcinogens[30]. In HCC, the downregulation of *CYP3A4* was regarded as an independent predictor for early recurrence and a suppressor associated with poor prognosis [31,32]. A previous study by our team showed that *CYP3A4* could facilitate the transformation of aflatoxin B1 to AFB1-exo-8,9-epoxide, therefore participating in the tumorigenesis of HCC[33]. In GO and KEGG analysis, *CYP3A4* was enriched to some carcinogenesis process like fatty acid metabolic process, monooxygenase activity, oxidoreductase activity and chemical carcinogenesis, which indicated the carcinogenic effect of *CYP3A4* in HCC. Participating in the HIF-1 pathway and influencing the secretion of VEGF, *CDC20* also played an important role in HCC progression[34]. Besides, GO and KEGG analysis also showed that *CDC20* might be involved in nuclear division and chromosome segregation which were largely correlated with carcinogenesis. In conclusion, *CYP3A4* and *CDC20* were closely associated with HCC development as the ACE2-related genes in HCC. However, few studies had reported the relation between *ACE2* and *CYP3A4* in HCC, and that might be a direction worthy of further study.

*ACE2* playing roles in HCC might be relevant to its classical molecular function – metallopeptidase activity. The expression of matrix metalloproteinase (MMP) 2 and MMP9 is closely related to different types of tumor metastasis. Non-small cell lung cancer cells have decreased invasion ability due to the inhibition of the expression of these two genes by *ACE2*, which reveals that *ACE2* as a zinc metalloprotease plays an important inhibitory role in cancer cells[35]. In our research, *ACE2* and the three *ACE2*-related genes – *NR3C2, MME*, and *MEP1B* – in the PPI subnetwork clustered simultaneously in the metallopeptidase activity function, which suggests that metallopeptidase activity may be one of the mechanisms by which *ACE2* plays a role in HCC. Regrettably, there is currently a lack of sufficient literature to prove this suppose, which is a direction worthy of experimental research.

There are some limitations in our study, such as insufficiency of samples for the prognosis analysis and the lack of in vitro experiments.

## Conclusion

To sum up, we confirmed the downregulation of *ACE2* in HCC and explored the potential clinical value and mechanisms of *ACE2* expression in HCC. *ACE2* expression was related to favorable prognosis, and it makes it feasible to distinguish HCC. Chemical carcinogenesis, catabolic process and the cell cycle were potential pathways that *ACE2* involved in HCC, and this needs to be further explored.
